# HSP90B1 facilitates glioma radiotherapy resistance by regulating RhoC ubiquitin‒proteasome degradation

**DOI:** 10.1016/j.gendis.2025.101756

**Published:** 2025-07-01

**Authors:** Jiacheng Xu, Yuduo Guo, Jingjing Yang, Guanjie Shang, Weihai Ning, Deshan Liu, Hongwei Zhang, Yongmei Song

**Affiliations:** a State Key Laboratory of Molecular Oncology, National Cancer Center/National Clinical Research Center for Cancer/Cancer Hospital, Chinese Academy of Medical Sciences and Peking Union Medical College, Beijing 100021, China; b Department of Neurosurgery, Sanbo Brain Hospital, Capital Medical University, Beijing 100093, China; c Department of Neurosurgery, Xuanwu Hospital, Capital Medical University/China International Neuroscience Institute (China-INI), Beijing 100053, China

**Keywords:** Glioma, Heat shock proteins, HSP90B1, Radiotherapy, RhoC

## Abstract

Gliomas are primary brain tumors known for their resistance to radiotherapy and frequent recurrence. This might result from the high heterogeneity and transcriptional plasticity of gliomas. Heat shock proteins are associated with unfavorable tumor outcomes and protect tumors from the effects of radiotherapy. However, their influence on brain tumors is not fully understood. Initial analyses of glioma patients from the Cancer Genome Atlas (TCGA) and the Chinese Glioma Genome Atlas (CGGA) databases who had undergone radiotherapy identified HSP90B1 as a crucial gene affecting patient prognosis. Subsequent investigations revealed that HSP90B1 enhanced the proliferation, migration, and invasion of glioma cells. It was also found to protect glioma cells from radiotherapy-induced apoptosis. Co-immunoprecipitation (CO-IP) found that HSP90B1 directly interacted with RhoC and protected it from degradation via the ubiquitin–proteasome pathway. Rescue experiments indicated that HSP90B1 might facilitate glioma migration, invasion, and radiotherapy resistance by modulating RhoC expression. A mouse model further demonstrated that gliomas expressing high levels of HSP90B1 exhibited decreased sensitivity to radiotherapy. Overall, our research revealed that HSP90B1 significantly impacts the prognosis of glioma patients treated with radiotherapy. Additionally, HSP90B1 might enhance glioma metastasis and resistance to radiotherapy by regulating RhoC expression. This regulatory effect was achieved by the directly binding of HSP90B1 to RhoC, thereby preventing its degradation through the ubiquitin–proteasome pathway.

## Introduction

Brain tumors, due to their location and locally invasive growth characteristics, exhibit high incidence and mortality rates.^1^ Secondary brain tumors, originating from metastases from extracranial cancers, are found to be five to ten times more prevalent than primary brain tumors, which originate from neoplasms within the brain itself.^2^ Gliomas, accounting for 30% of all primary brain tumors and 80% of malignant ones, were the predominant type, significantly contributing to mortality among these patients.^3^ Following the World Health Organization's guidelines for classifying central nervous system tumors, glioblastomas are recognized as the most prevalent malignant tumors, accounting for 14.2% of all such tumors and 50.9%^1^ of malignant types. The one-year relative survival rate was recorded at 42.9%, while the five-year rate was only 6.9%.^3^ The standard treatments include surgical removal followed by radiotherapy (RT) and chemotherapy.^4^, ^5^, ^6^ However, due to the heterogeneity and transcriptional plasticity of tumors, radiotherapy resistance and recurrence were inevitable.^7^ Although various approaches, such as chemotherapy, radiotherapy, targeted agents, and immunotherapies, have led to certain advancements in glioma management, overcoming radioresistance remains a critical and unresolved challenge in the field.^8^ Therefore, it is crucial to develop new molecular targeting strategies to combat radiotherapy resistance.

Heat shock proteins (HSPs) participate in protein folding, transport, and degradation, functioning as molecular chaperones.^9^ HSPs are crucial for the survival, migration, invasion, angiogenesis, apoptosis, and drug resistance of tumor cells,^10^, ^11^, ^12^, ^13^ closely associated with tumor progression. Notably, under extreme conditions such as high temperatures, drug exposure, or other stimuli that could induce protein denaturation, HSPs are able to promote protein folding and maintain their normal structure and function.^14^^,^^15^ This observation prompted further inquiry into the role of HSPs in conferring radiotherapy resistance in gliomas.

Studies have shown that HSPs are associated with poor prognosis and radiotherapy resistance in tumors. Compared to normal cells, tumor cells express higher levels of heat shock protein 70 (HSP70), which induces radiotherapy resistance.^16^^,^^17^ Suppressing HSP70 expression significantly enhances radiotherapy-induced tumor cell apoptosis, thereby increasing radiotherapy sensitivity.^18^ Heat shock protein 27 (HSP27) was also found to be associated with radiotherapy resistance in tumor cells.^19^ HSP27 increases resistance to oxidative stress in tumor cells through glucose-6-phosphate dehydrogenase-dependent mechanisms and antioxidant activity.^20^ Knocking out the HSP27 gene enhances the sensitivity to radiotherapy in head and neck squamous cell carcinoma(HNSCC) and prostate cancer cells.^21^ Additionally, heat shock protein 90 (HSP90) is recognized for its therapeutic relevance in cancer management. Inhibiting HSP90 increases the sensitivity of tumor cells to radiotherapy and reduces their proliferation.^22^^,^^23^ These highlight the substantial potential of inhibiting HSPs to increase tumor radiosensitivity. However, the influence of HSPs on glioma is not yet fully understood.^24^ Therefore, we intended to identify and examine the specific HSP gene that plays a role in radiotherapy resistance in gliomas and explore the regulatory mechanism, aiming to identify potential adjuvant treatments for glioma.

In this study, we initially identified heat shock protein 90 beta family member 1 (HSP90B1) in glioma patients treated with radiotherapy, which influenced their prognosis. We revealed that HSP90B1 could enhance the proliferation and migratory and invasive capabilities of tumor cells. Additionally, our analysis indicated that HSP90B1 could protect tumor cells from radiotherapy-induced apoptosis, likely due to its direct binding to ras homolog family member C (RhoC), thereby preventing its degradation via the ubiquitin‒proteasome pathway. Animal experiments also revealed that tumors with high HSP90B1 expression exhibited greater resistance to radiotherapy.

## Materials and methods

### Data collection and analysis

RNAseq data, along with matching clinical details for both low-grade and high-grade gliomas, were obtained from the Cancer Genome Atlas (TCGA) database at https://portal.gdc.cancer.gov. Furthermore, we sourced both glioma sequencing data and related clinical details from the Chinese Glioma Genome Atlas (CGGA) database, accessible at https://www.cgga.org.cn. We initially identified patients who had undergone radiotherapy and arranged these patients by survival time. Based on the mean survival time, we categorized patients into short-term survival (STS) and long-term survival (LTS) groups. Patients whose survival time less than the mean but who remained alive during the observation period were independently classified into the LTS group. Subsequently, we conducted background correction on the glioma RNA sequencing data, removed missing data, and normalized the datasets. Differential analysis of RNA sequencing data between the groups was performed using RStudio software and the DESeq2 package. We intersected the differentially expressed genes (DEGs) analyzed from both the TCGA and CGGA databases and selected HSP genes from among them.

### Clinical sample collection and processing

The glioma samples were pathologically diagnosed as Grade II, III, or IV gliomas. The glioma and normal brain tissue samples used in this study were all sourced from Sanbo Brain Hospital, Capital Medical University. After surgical removal, the tumors were quickly submerged in liquid nitrogen for a duration of half an hour, followed by relocation to a −80 °C environment for extended preservation. Approval for this study was granted by the Ethics Committee of Sanbo Brain Hospital, Capital Medical University. Ethical approval and participant consent were obtained prior to study initiation.

### Immunohistochemistry

The paraffin-embedded specimens were baked in an oven for 6 h, followed by dewaxing and rehydration. After blocking endogenous peroxidases with a blocking agent for 20 min, the sections were treated with ethylenediaminetetraacetic acid (EDTA) antigen retrieval solution. Following blocking with sheep serum, the sections were subsequently placed in a cold environment at 4 °C to react overnight with the primary antibody, which had been suitably thinned. The sections were then washed with phosphate buffered saline (PBS) to remove residual antibodies and treated with an enhancer solution for 20 min, followed by incubation with secondary antibody. Diaminobenzidine staining was employed to visualize the reaction under a microscope, which was halted upon achieving the desired color development. The sections were counterstained with hematoxylin, subsequently treated with hydrochloric acid and ethanol for differentiation, and enhanced with ammonia water for bluing. Ultimately, the sections underwent dehydration through a graded series of alcohols and were encapsulated in neutral resin for subsequent microscopic analysis and scoring.

### Quantitative real-time PCR (qRT‒PCR)

Following the respective user manual, we isolated and purified total cellular RNA using the RNAExpress Total RNA Kit (NCM Biotech). With the Promega reverse transcription kit, we synthesized cDNA from mRNA through reverse transcription reactions. The CFX96 Real-Time System (Bio-Rad) was employed to quantify mRNA expression. After normalization to actin, the expression of specific genes was determined via the 2^-ΔΔCT^ method. Table S1 details the gene primers applied in our experiments.

### Cell culture and transfection

From the State Key Laboratory of Molecular Oncology, we acquired the glioma cell lines LN229, U87, and U343 for this research. Cultured in dulbecco's modified eagle's medium (DMEM) enriched with 10% fetal bovine serum and antibiotics, the cells were maintained at the optimal conditions of 37 °C and 5% CO_2_.

According to the manual, the double-stranded siRNAs were transfected into cells with the assistance of Lipofectamine 2000. The overexpression plasmids required NEOFECT for successful transfection into cells. To establish cell lines with stable knockdown or overexpression of HSP90B1, we employed lentiviral infection. The knockdown lentiviral vector component sequence was Ubi-MCS-firefly_Luciferase-IRES-Puromycin, and the overexpression lentiviral vector component sequence was pcSLenti-EF1-Luc-F2A-Puro-CMV-MCS-WPRE. Tables S2 and S3 detail the sequences for the siRNAs and shHSP90B1 utilized in this research.

### Protein extraction and Western blot

The cells were lysed on ice using RIPA buffer supplemented with protease and phosphatase inhibitors. After centrifugation, the supernatant containing the total cellular protein was denatured with 2-mercaptoethanol. We conducted SDS‒PAGE to separate proteins of various molecular weights, which were then placed onto a polyvinylidene fluoride (PVDF) membrane. Subsequently, we treated the membrane with 5% non-fat milk, employing 5% BSA specifically for phosphorylated proteins. Subsequently, the membrane underwent incubation at 4 °C, treated with a properly diluted primary antibody overnight. The following day, the excess primary antibody was removed by washing with PBS with Tween-20 (PBST), and the membrane was subsequently incubated with a horseradish peroxidase–linked secondary antibody. An enhanced chemiluminescence (ECL) kit revealed protein expression, and images were subsequently captured with the Image800-3 system.

### Reagents and antibodies

Tables S4 and S5 catalog the reagents and antibodies employed throughout this study.

### Cell proliferation

Using a colony formation assay and a CCK8 assay, we assessed cell proliferation. For the colony formation assay, we distributed 2000 cells from each group into six-well plates and allowed them to grow for 10 days. After fixing the colonies with methanol and staining them with crystal violet, we counted the cell clusters for statistical analysis.

For the CCK8 assay, each well of a 96-well plate received 2000 cells, with subsequent cell counts taken at 24, 48, 72, and 96 h. Following the manual's instructions, we introduced 10 μl of CCK8 solution into each well. After allowing the solution to react at 37 °C for 1 h, we then determined the optical density at 450 nm.

### Cell migration and invasion assays

For the migration and invasion experiments, 30,000 cells were placed in the upper chamber of the Transwell plate with serum-free DMEM, while the lower chamber was filled with complete DMEM. For the invasion assay, the upper chamber additionally received serum-free DMEM containing 2% Matrigel matrix. After 24 h, methanol was added to fix the cells, followed by crystal violet staining. Microscopic observation and cell counting were conducted to statistically analyze the differences between the groups.

### Immunoprecipitation

Cells were lysed in lysis buffer (137 mM NaCl, 20 mM Tris-HCl [pH = 8], 1% NP-40, 2 mM EDTA) containing protease inhibitors. Antibodies were added to the supernatant obtained by centrifugation, and the mixture was incubated at 4 °C overnight. Following incubation with Protein A/G PLUS-Agarose, the immune complexes were subsequently subjected to analysis via Western blot.

### Immunofluorescence

Paraformaldehyde was applied to fix the cells, followed by permeabilization with 0.5% Triton X-100. Following sheep serum blocking, the primary antibody, which was suitably diluted, was applied and incubated at 4 °C overnight. Afterward, a fluorescently labeled secondary antibody was applied in the dark, and DAPI (4′,6-diamidino-2-phenylindole) was used for nuclear staining. The fluorescent signals were observed by selecting the appropriate excitation wavelengths and examining the samples under a fluorescence microscope.

### Apoptosis assay

Cells from both the supernatant and adherent layers were harvested, rinsed twice with PBS, and then suspended in binding buffer. Annexin V-FITC or Annexin V-APC dye along with propidium iodide (PI) dye were added and thoroughly mixed. The mixture was incubated in the dark for 5–10 min. Apoptotic cells were quantified via flow cytometry, and statistical analyses were conducted to evaluate differences among the groups.

### Radiotherapy

In cell experiments, LN229 cells were subjected to 10 Gy of irradiation, while U87 cells received 15 Gy. Two days after irradiation, cell apoptosis was assessed via flow cytometry, and the indicated proteins were detected through Western blot. In animal experiments, mice underwent radiotherapy at a dose of 3.3 Gy per session, with treatments administered every two days for a total of three sessions.^25^^,^^26^

### Xenograft in nude mice

Suspensions of 3 × 10^6^ cells were injected into the hind legs of 5-week-old female athymic nude mice via subcutaneous administration. Once the tumors grew to around 25 mm^3^ in volume, localized radiation therapy was administered to the tumor area. Calipers were employed to measure tumor length (L) and width (W), and the volume was determined by formula (L × W^2^)/2.

### Statistical analysis

To evaluate intergroup variation, we applied Student *t*-test, with a *p* value less than 0.05 considered statistically significant. For differential analysis, the selection criteria were set as |log2FoldChange| ≥ 0.4 and a false discovery rate (FDR) < 0.01.

## Results

### High HSP90B1 expression linked to poor prognosis in radiotherapy-treated glioma patients

**Figure 1:**
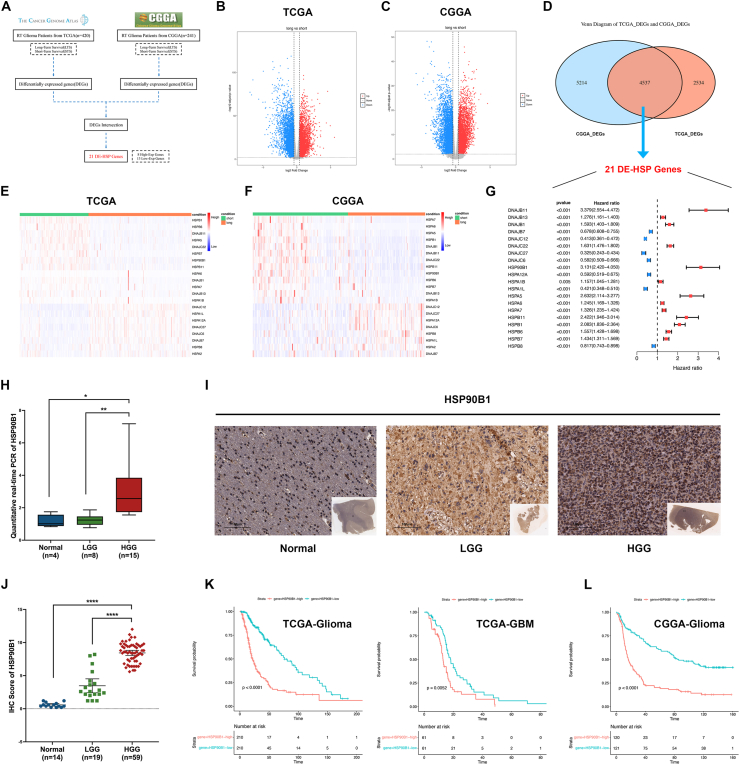
Selection of heat shock protein genes that affect the prognosis of glioma patients receiving radiotherapy. **(A)** Flowchart for the selection of heat shock proteins related to poor prognosis in glioma patients who had undergone radiotherapy. **(B, C)** Gene expression differences between LTS and STS groups in glioma patients who had received radiotherapy were analyzed in the TCGA (B) and CGGA (C) databases. **(D)** Differentially expressed genes from the TCGA and CGGA databases were intersected, and HSP genes with differential expression were extracted. **(E, F)** Heatmap analysis of the expression of differentially expressed HSPs in the TCGA (E) and CGGA (F) databases. **(G)** Univariate Cox analysis of the hazard ratio of HSP genes on the survival of patients who had undergone radiotherapy. **(H)** qPCR analysis of HSP90B1 mRNA expression levels in normal brain tissues (*n* = 4), low-grade gliomas (*n* = 8), and high-grade gliomas (*n* = 15). **(I, J)** Immunohistochemical analysis of HSP90B1 protein expression levels in normal brain tissues (*n* = 14), low-grade gliomas (*n* = 19), and high-grade gliomas (*n* = 59). **(K)** Assessment of HSP90B1 expression and its link to prognosis in glioma patients (left) who received radiotherapy and even in glioblastoma patients (right) who received radiotherapy from the TCGA database. **(L)** HSP90B1 expression levels were evaluated in relation to patient prognosis for glioma cases treated with radiotherapy based on data from the CGGA database.

To further analyze the factors contributing to radiotherapy resistance in glioma (Fig. 1A), we initially acquired RNA sequencing data along with clinical information for glioma patients receiving radiotherapy from the TCGA and CGGA databases. The TCGA dataset included 420 patients and the CGGA dataset included 241 patients who had received radiotherapy. We then categorized all patients into two groups based on the mean survival time and survival status: long-term survival (LTS), short-term survival (STS). In the TCGA dataset, 252 patients were assigned to the LTS group and 168 to the STS group. In the CGGA dataset, 108 patients were assigned to the LTS group and 133 to the STS group. Subsequently, we performed differential expression analysis between the LTS and STS groups using the DESeq2 package (Fig. 1B, C). From the TCGA dataset, we identified 3398 up-regulated and 3673 down-regulated genes, while the CGGA dataset revealed 4653 up-regulated and 5098 down-regulated genes. Considering the intersection of the DEGs between the TCGA and CGGA datasets, we obtained 4537 DEGs (Fig. 1D). Heat shock proteins regulated tumor cell survival, protecting them from radiotherapy-induced cell death.^27^^,^^28^ Therefore, based on the description of HSPs in Zhang's study,^29^ we identified 21 HSP genes among the 4537 DEGs, including 8 up-regulated and 13 down-regulated genes (Fig. 1D). The heatmap distinctly illustrated how these 21 HSP genes varied among the different groups (Fig. 1E, F). To identify genes that might contribute substantially to radiotherapy resistance in glioma, we evaluated the hazard ratio (HR) for each of the 21 HSP genes concerning patient survival after radiotherapy (Fig. 1G). Among these genes, HSPA2 was excluded due to its lack of significance in univariate Cox analysis (Fig. 1G). Although DNAJB11 exhibited the highest hazard ratio, previous studies have indicated that HSP90 family members might contribute to the development of radioresistance.^30^ Therefore, HSP90B1 was selected for further investigation due to its high HR (HR = 3.131) and the limited understanding of its function in glioma.

Firstly, we conducted qPCR and immunohistochemistry to examine HSP90B1 expression in gliomas. HSP90B1 exhibited elevated expression in glioma according to RNA and protein analyses (Fig. 1H–J). Furthermore, HSP90B1 levels were elevated in high-grade glioma (HGG) (Fig. 1J), indicating a significant association with glioma malignancy. Next, we performed survival analysis to verify the impact of HSP90B1 on patients receiving radiotherapy. Patients exhibiting high HSP90B1 expression in both the TCGA and CGGA cohorts had worse prognoses (Fig. 1K, L). Importantly, high HSP90B1 expression was further linked to unfavorable outcomes in WHO grade IV glioblastoma patients receiving radiotherapy (Fig. 1K). These findings indicate that HSP90B1 is critically involved in radiotherapy resistance in glioma, motivating us to further explore its function.

### HSP90B1 promoted glioma cell proliferation, migration, and invasion capabilities

**Figure 2:**
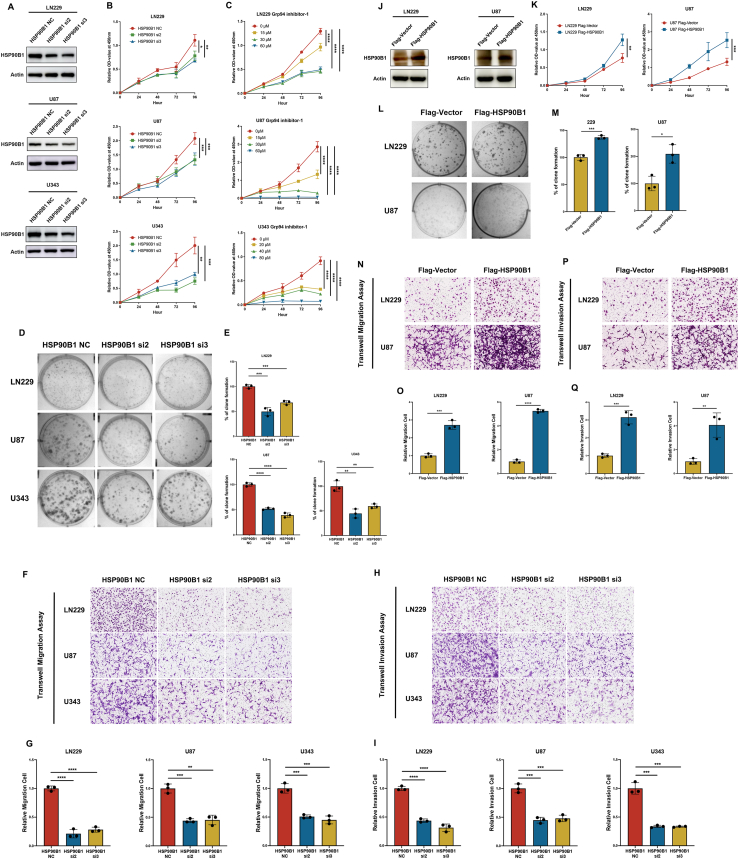
HSP90B1 promoted glioma cell proliferation, migration and invasion. **(A)** Knockdown efficiency of HSP90B1 in LN229, U87, and U343 cells. **(B)** Cell proliferation after HSP90B1 knockdown was assessed via the CCK8 assay. **(C)** The effects of different concentrations (1/2 IC_50_, IC_50_, and 2 × IC_50_) of the HSP90B1 inhibitor (Grp94 inhibitor-1) on cell proliferation were evaluated via the CCK8 assay. **(D, E)** Colony formation assay was used to analyz the effects of HSP90B1 knockdown on cell proliferation. **(F–I)** HSP90B1 knockdown-associated changes in cell migration and invasion were quantified via Transwell analysis. **(J)** The successful overexpression of HSP90B1 in cells was validated through Western blot analysis. **(K)** Cell proliferation affected by HSP90B1 overexpression was evaluated with the CCK8 assay. **(L, M)** Colony formation was analyzed to determine the effect of HSP90B1 overexpression. **(N**–**Q)** Cell migration and invasion were examined to assess the influence of HSP90B1 overexpression, with Transwell assays applied for analysis.

To investigate HSP90B1's role in the glioma malignant progression, we knocked down HSP90B1 expression in three glioma cell lines (LN229, U87, and U343) (Fig. 2A) and assessed cell proliferation using the CCK8 assay. We found that HSP90B1 knockdown significantly reduced cell proliferation (Fig. 2B). Similarly, based on the IC50 values of different cells (Fig. S1A–C), when we added the HSP90B1 inhibitor Grp94 inhibitor-1 to the culture supernatant, cell proliferation was also significantly inhibited, with the inhibitory effect becoming more pronounced as the inhibitor concentration increased (Fig. 2C). Furthermore, the results of the colony formation assays indicated that knocking down HSP90B1 substantially hindered the cellular colony-forming ability (Fig. 2D, E). In the transwell assay, the migration and invasion in HSP90B1 knockdown cells were notably reduced (Fig. 2F–I).

Conversely, when we overexpressed HSP90B1 in glioma cells (Fig. 2J), we found that cells with high HSP90B1 expression exhibited enhanced proliferation and colony formation abilities (Fig. 2K–M). Additionally, HSP90B1 overexpression significantly increased both cell migration and invasion (Fig. 2N–Q). Overall, HSP90B1 was identified as a major regulator of glioma cell growth and metastasis.

### HSP90B1 regulated the resistance of gliomas to radiotherapy-induced apoptosis

**Figure 3:**
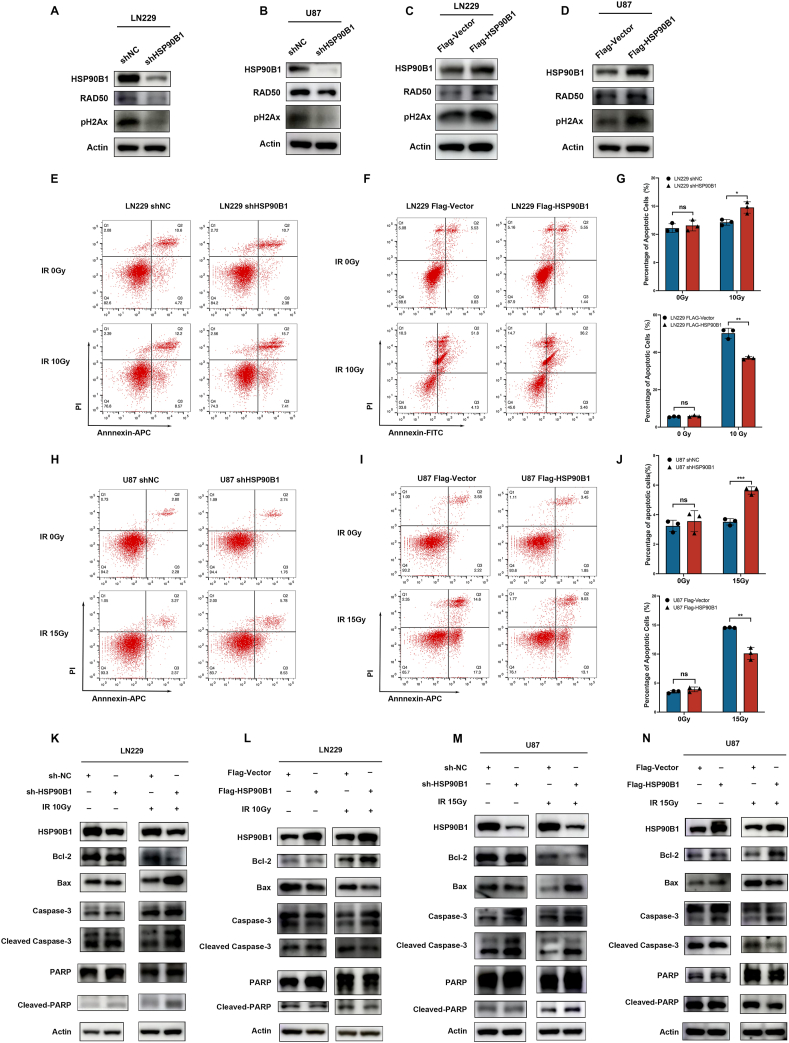
HSP90B1 inhibited radiotherapy-induced apoptosis. **(A**–**D)** Western blot was used to examine the effects of HSP90B1 knockdown (A, B) and overexpression (C, D) on the expression of DNA repair regulatory factors (RAD50 and pH2Ax). **(E**–**G)** Flow cytometry was used to assess apoptosis in LN229 cells with HSP90B1 knockdown (E) or overexpression (F) following radiotherapy (10 Gy) or without treatment. **(H**–**J)** Flow cytometry was used to analyze apoptosis in U87 cells with HSP90B1 knockdown (H) or overexpression (I) after radiotherapy (15 Gy) or without treatment. **(K, L)** Western blot monitored changes in the apoptotic regulators (Bcl-2, Bax, cleaved caspase-3, and cleaved-PARP) expression in LN229 cells with HSP90B1 knockdown (K) or overexpression (L) after radiotherapy or without treatment. **(M, N)** Western blot evaluated changes in the apoptotic regulators (Bcl-2, Bax, cleaved caspase-3, cleaved-PARP) expression in U87 cells with HSP90B1 knockdown (M) or overexpression (N) following radiotherapy or without treatment.

DNA damage repair is crucial in mediating the radiotherapy resistance of tumor cells.^31^ To analyze the potential of HSP90B1 in regulating radiotherapy resistance in glioma cells, we first investigated whether HSP90B1 could regulate DNA damage repair mechanisms. pH2Ax and RAD50, key regulators of DNA repair, are crucial for recognizing and fixing DNA double-strand breaks (DSBs).^32^, ^33^, ^34^ After knocking down HSP90B1 in the LN229 and U87 cell lines, we observed a corresponding down-regulation of pH2Ax and RAD50 expression (Fig. 3A, B). Conversely, in glioma cells overexpressing HSP90B1, the expression of pH2Ax and RAD50 was correspondingly up-regulated (Fig. 3C, D). These initial results suggested that HSP90B1 was involved in DNA repair and radiotherapy response in gliomas.

To investigate HSP90B1's involvement in radiotherapy resistance in gliomas, we subjected glioma cells to radiation therapy and analyzed its impact on cell apoptosis. Flow cytometry analysis showed that knocking down HSP90B1 in LN229 cells did not significantly affect apoptosis. However, following radiation treatment (10 Gy), apoptosis significantly increased in the HSP90B1 knockdown group (Fig. 3E, G). Conversely, although the overexpression of HSP90B1 had no significant effect on apoptosis, after radiation treatment, apoptosis was significantly lower in glioma cells overexpressing HSP90B1 than in the control group (Fig. 3F, G). These indicated that HSP90B1 might protect glioma cells from radiotherapy-induced cell death, a finding that was similarly confirmed in the U87 cell line (Fig. 3H–J).

To further confirm the effect of HSP90B1 on apoptosis following radiotherapy, we examined changes in different regulatory molecules involved in the apoptosis process. Compared to the control group, post-radiation treatment, LN229 cells with HSP90B1 knockdown exhibited a significant reduction in Bcl-2 (anti-apoptotic gene), while Bax (pro-apoptotic gene), cleaved caspase-3, and cleaved-PARP levels were elevated, indicating increased apoptosis (Fig. 3K). Conversely, in LN229 cells overexpressing HSP90B1, post-radiation treatment elevated Bcl-2 levels while reducing Bax, cleaved caspase-3, and cleaved-PARP expression, indicating reduced apoptosis (Fig. 3L). The same trends were observed in the U87 cell line (Fig. 3M, N). However, for cells not subjected to radiation treatment, no significant changes in regulatory molecules involved in apoptosis were observed, regardless of whether HSP90B1 was knocked down or overexpressed (Fig. 3K–N). Overall, HSP90B1 appeared to reduce radiotherapy-induced apoptosis in glioma cells.

### HSP90B1 regulated the expression of RhoC through the ubiquitin–proteasome pathway

**Figure 4:**
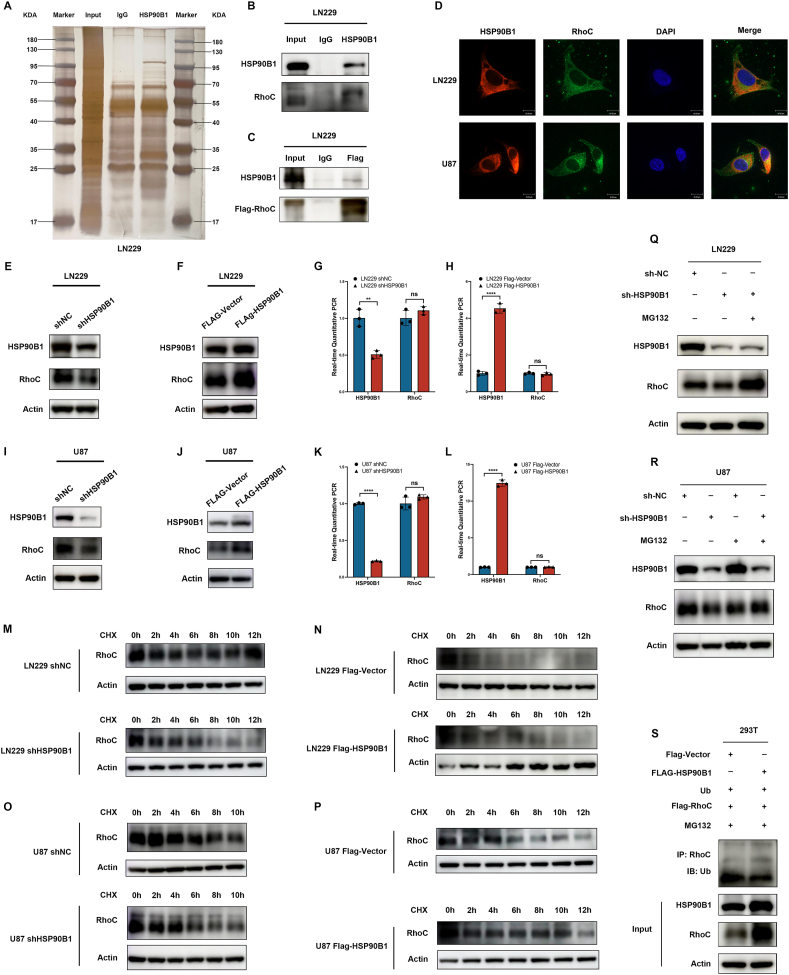
HSP90B1 interacted with RhoC and regulated its ubiquitin–proteasome degradation pathway. **(A)** Proteins bound to HSP90B1 were immunoprecipitated followed by silver staining of the protein samples. (**B)** Co-immunoprecipitation (CO-IP) analysis was performed to investigate the interaction of HSP90B1 with RhoC. An anti-HSP90B1 antibody was employed to immunoprecipitate HSP90B1, while RhoC CO-IP was identified through Western blot analysis. The input confirmed total protein expression, and an IgG control was used to confirm specificity. **(C)** CO-IP analysis of the interaction between RhoC and HSP90B1 was performed. Flag-tagged RhoC, overexpressed in LN229 cells, was immunoprecipitated using an anti-Flag antibody. **(D)** Immunofluorescence analysis of the colocalization of HSP90B1 and RhoC in LN229 and U87 cells was performed. The cells were stained with anti-HSP90B1 (red) and anti-RhoC (green) antibodies, with DAPI (blue) marking the nuclei. Merged image showing significant colocalization of HSP90B1 and RhoC in the cytoplasm (yellow). **(E**–**H)** RhoC protein and mRNA levels were measured in LN229 cells following HSP90B1 knockdown (E, G) or overexpression (F, H). **(I**–**L)** In U87 cells, RhoC protein and mRNA levels were evaluated after HSP90B1 knockdown (I, K) or overexpression (J, L). **(M, N)** CHX (50 μg/mL) was added to the culture supernatant of LN229 cells, and protein samples collected at various times were examined via Western blot to assess the impact of knocked down (M) or overexpressed (N) HSP90B1 on RhoC protein degradation, with actin serving as an internal control. **(O, P)** CHX (50 μg/mL) was added to the culture supernatant of U87 cells, and protein samples collected at different times were examined via Western blot to assess the effect of knocked down (O) or overexpressed (P) HSP90B1 on RhoC protein degradation. **(Q, R)** RhoC expression in LN229 (Q) and U87 (R) cells with knocked down HSP90B1 was assessed by Western blot after treatment with MG132 (10 μM) or without MG132 treatment, with actin serving as an internal control. **(S)** In 293T cells overexpressing HSP90B1, RhoC, or Ub, the cells were treated with MG132. Immunoprecipitation using an anti-RhoC antibody and Western blot analysis were used to assess the ubiquitination levels of RhoC.

To further elucidate the mechanisms through which HSP90B1 modulates radiotherapy resistance in glioma cells. By conducting (CO-IP) experiment and subsequent liquid chromatography–mass spectrometry(LC–MS) analysis, we identified proteins that interact directly with HSP90B1. Among all the bound proteins, we specifically noted a binding interaction between HSP90B1 and RhoC (Fig. 4A). A related study on cervical cancer indicated that RhoC is involved in modulating tumor radioresistance, mediated by proteins associated with the DNA damage repair pathway.^35^ These findings led us to postulate that HSP90B1 could influence glioma radioresistance through its interaction with RhoC. To validate this hypothesis, we first performed a CO-IP assay to confirm the LC–MS results, demonstrating that HSP90B1 directly interacted with RhoC (Fig. 4B). Conversely, when we overexpressed Flag-tagged RhoC in LN229 cells, RhoC was also found to directly interact with HSP90B1 (Fig. 4C), suggesting a mutual interaction between HSP90B1 and RhoC. Furthermore, immunofluorescence assays demonstrated the co-localization of HSP90B1 and RhoC in glioma cells (Fig. 4D).

Additionally, we investigated whether HSP90B1's interaction with RhoC extends beyond mere binding to include the modulation of RhoC expression. Alterations in HSP90B1 levels, through either knockdown or overexpression in LN229 cells, led to respective decreases and increases in RhoC protein levels (Fig. 4E, F), without affecting RhoC mRNA levels (Fig. 4G, H). These observations were replicated in U87 cells (Fig. 4I–L), suggesting that HSP90B1 might regulate RhoC expression more at the protein level than at the mRNA level.

Under typical conditions, potential oncogenic alterations such as mutations or overexpression, which are not translatable in the absence of heat shock proteins due to insufficient chaperone activity, would be degraded through the proteasome-mediated pathway. However, elevated levels of HSPs could stabilize such oncogenic proteins, preventing their degradation and thus promoting tumor proliferation and transformation.^12^^,^^36^^,^^37^ Based on these insights, we theorized that HSP90B1 might modulate RhoC expression by preventing its degradation. To test this theory, we treated cell culture supernatants with the protein synthesis inhibitor cycloheximide (CHX) and monitored RhoC protein degradation at various intervals (0, 2, 4, 6, 8, 10, and 12 h). Our results revealed that the RhoC protein underwent earlier degradation following HSP90B1 knockdown in LN229 cells (Fig. 4M), whereas degradation was significantly delayed following HSP90B1 overexpression (Fig. 4N). This pattern was also confirmed in U87 cells (Fig. 4O, P), indicating that HSP90B1 might increase RhoC expression by inhibiting its protein degradation.

Protein degradation in eukaryotic cells primarily occurred through two distinct mechanisms: the ubiquitin–proteasome system and lysosome-mediated autophagy. Of these, the ubiquitin–proteasome system served as the main route for protein catabolism, accounting for the degradation of over 80% of cellular proteins.^38^, ^39^, ^40^, ^41^ To elucidate the pathway through which HSP90B1 modulated the degradation of the RhoC protein, we initially introduced the proteasome inhibitor MG132 into the culture supernatant of cells with HSP90B1 knockdown. Without MG132 treatment, the knockdown of HSP90B1 led to a notable reduction in RhoC protein levels. Conversely, treatment with MG132 restored the protein levels of RhoC in these cells (Fig. 4Q, R). Additionally, a significant reduction in RhoC ubiquitination was observed in HSP90B1-overexpressing cells after MG132 treatment (Fig. 4S). Collectively, these findings suggest that HSP90B1 protect RhoC from ubiquitin–proteasome pathway-mediated degradation by directly interacting with RhoC, thereby increasing RhoC expression.

### HSP90B1 modulated the radiotherapy resistance and migratory and invasive capabilities of glioma cells through RhoC

**Figure 5:**
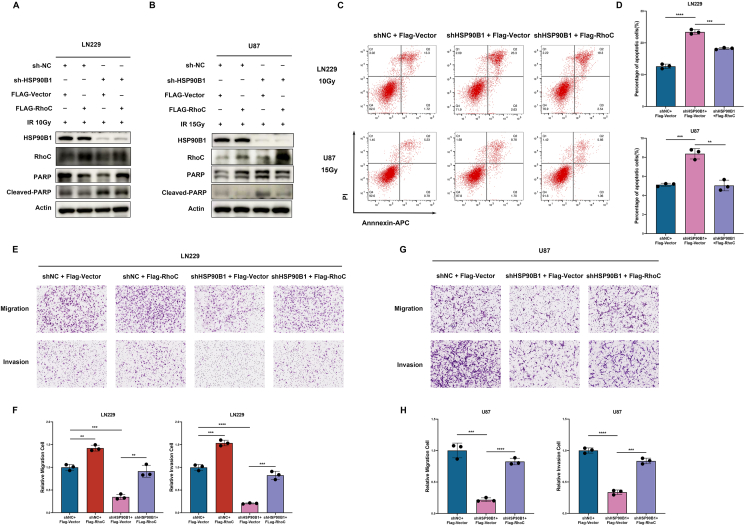
HSP90B1 promoted glioma radioresistance and migratory and invasive capabilities by regulating RhoC expression. **(A, B)** LN229 (A) or U87 (B) cell was divided into four groups: control, HSP90B1 knockdown, RhoC overexpression control, and HSP90B1 knockdown combined with RhoC overexpression. Two days after radiotherapy, apoptosis marker expression was analyzed via Western blot. **(C, D)** Flow cytometry was used to assess the impact of HSP90B1 knockdown and combined knockdown of HSP90B1 with overexpression of RhoC on cell apoptosis following radiotherapy. **(E**–**H)** Through Transwell assays, the effects of HSP90B1 knockdown and combined knockdown of HSP90B1 with overexpression of RhoC on the migratory and invasive capabilities of LN229 (E, F) and U87 (G, H) cells were evaluated.

Next, we sought to further investigate whether HSP90B1 exerts its effects by regulating the expression of RhoC. Following the radiotherapy treatment of glioma cells, cleaved-PARP expression was elevated in HSP90B1-knockdown glioma cells. However, the overexpression of RhoC in these HSP90B1-knockdown cells restored the levels of cleaved-PARP (Fig. 5A, B). Similarly, flow cytometry revealed more apoptotic HSP90B1-knockdown cells following radiotherapy, but a reduction in apoptosis was noted when RhoC was overexpressed in these cells (Fig. 5C, D). These findings indicate that HSP90B1 promotes radiotherapy resistance in gliomas by regulating RhoC expression.

Additionally, in cells with HSP90B1 knockdown, we observed a reduction in migration and invasion. However, when RhoC was overexpressed in these HSP90B1-knockdown cells, their migratory and invasive capabilities were restored (Fig. 5E–H). Taken together, these findings indicate that HSP90B1 enhances the radiotherapy resistance and migratory and invasive capabilities of glioma cells by modulating RhoC expression.

### HSP90B1 promoted the proliferation and radiotherapy resistance of gliomas in mice

**Figure 6:**
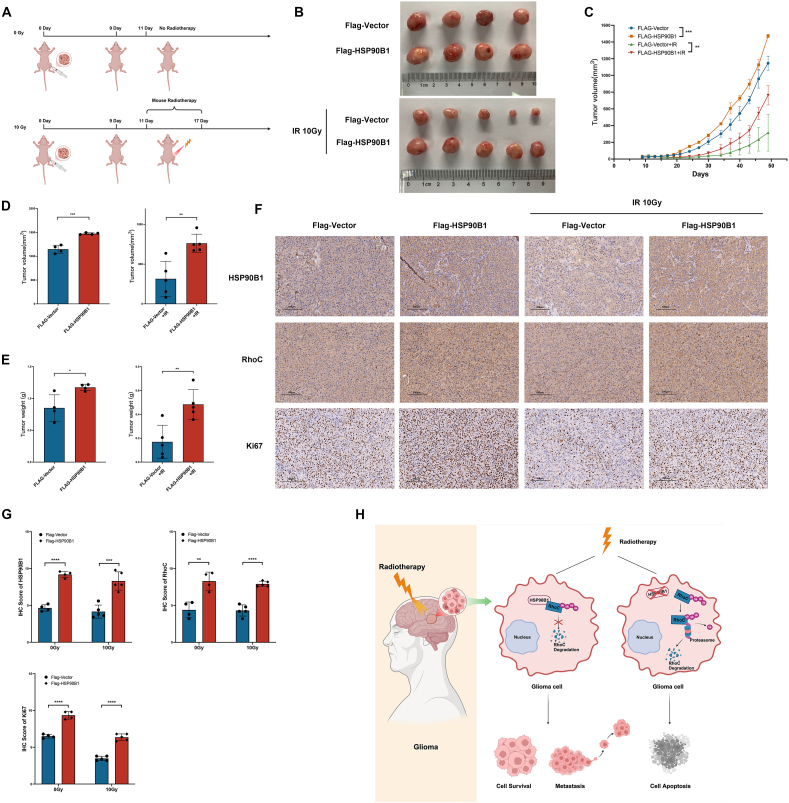
HSP90B1 enhanced tumor proliferation and radioresistance in mice. **(A)** The experiment was divided into four groups: Flag-Vector, Flag-HSP90B1, Flag-Vector + IR, and Flag-HSP90B1 + IR. Tumor cells from the different treatment groups were implanted subcutaneously into the mice, and the IR groups received radiotherapy after tumor formation. Tumor volume and mouse weight were regularly monitored, and at the conclusion of the experiment, tumor samples were gathered for further analysis. **(B, C)** Images of tumors (B) and tumor growth curves (C) from the different treatment groups. **(D, E)** Upon completion of the experiment, tumor volume (D) and tumor weight (E) were measured, and differences between groups were analyzed. **(F, G)** Immunohistochemistry was employed to examine HSP90B1, RhoC, and Ki67 expression across the various treatment groups. **(H)** A model illustrating the function of HSP90B1 in regulating radiotherapy resistance in glioma cells by inhibiting the degradation of RhoC.

Next, we sought to further explore whether HSP90B1 could enhance the radiotherapy resistance of gliomas in mice. As shown in Figure 6A, we first implanted glioma cells subcutaneously into nude mice and began localized radiotherapy when the tumor volume reached approximately 25 mm^3^. The first session of radiotherapy was conducted on day 11, followed by treatments every other two days, with each radiation dose being 3.3 Gy, for a total of three sessions. We observed that (Fig. 6B, C) although radiotherapy significantly inhibited tumor growth, tumors with high HSP90B1 expression grew faster than those in the control group did. Upon examining the final tumor volume and weight, we found that while HSP90B1 overexpression already promoted tumor growth in mice not subjected to radiotherapy, it exhibited a more pronounced pro-tumorigenic effect on irradiated mice (Fig. 6D, E). Additionally, monitoring of mouse body weight revealed a gradual decrease following radiotherapy, which then returned to normal levels (Fig. S2A). The body weight remained stable compared with that of the control group. Immunohistochemical analysis of Ki67 revealed that tumors with high expression of both HSP90B1 and RhoC showed significantly elevated Ki67 levels. Although Ki67 expression decreased following radiotherapy, it remained high in tumors with elevated HSP90B1 expression (Fig. 6F, G). These results indicate that HSP90B1 promotes tumor proliferation in mice and enhances their resistance to radiotherapy.

Overall (Fig. 6H), HSP90B1 showed elevated expression in glioma patients, promoting glioma cell proliferation, metastasis, and resistance to radiotherapy-induced apoptosis. Notably, HSP90B1 likely contributed to metastasis and radiotherapy resistance in glioma cells by inhibiting the ubiquitin–proteasome pathway degradation of RhoC.

## Discussion

Existing literature suggest that tumor cells exhibit heterogeneity in their response to radiotherapy, with some cancer cell subgroups possessing radiotherapy resistance,^42^ potentially due to heat shock protein-mediated resistance.^43^ While HSPs’ function in mediating radiotherapy resistance in other tumors had been identified, their impact on brain tumors remained unclear.^24^ To explore the HSPs influencing glioma radioresistance, we initially conducted a comprehensive analysis of patients from the TCGA and CGGA datasets who had undergone radiotherapy, identifying that HSP90B1 plays a significant role in glioma radioresistance. Although inhibiting HSP70 or HSP90 has been found to enhance radiotherapy-induced cytotoxic effects^22^^,^^23^^,^^44^^,^^45^, the role of HSP90B1 in this context has not been explored.

Our findings confirmed elevated levels of HSP90B1 in gliomas, which enhanced their proliferation and metastatic capabilities. Furthermore, HSP90B1 likely protected glioma cells from radiotherapy-induced apoptosis by inhibiting the ubiquitin–proteasome pathway degradation of RhoC. Research in other tumors has revealed the potential of enhancing tumor radiosensitivity by inhibiting HSPs. For example, HSP27, which belongs to the small heat shock protein group (molecular weight 12–43 kDa), underwent functional regulation through stress-induced phosphorylation at Ser-15, Ser-78, and Ser-82.^46^^,^^47^ Suppressing HSP27 notably boosted the vulnerability of human breast cancer cells to both UVC radiation-induced DNA damage and interferon (IFN)-mediated cytotoxic effects.^48^ Moreover, HSP27 down-regulation in HNSCC notably promoted apoptosis in response to radiation, inhibited colony formation, and deactivated Akt signaling.^21^ For other members, inhibition of HSP70 was shown to provoke apoptosis in tumors and enhance their sensitivity to radiation.^45^ After cellular damage from ionizing radiation, HSP70 enters the tumor microenvironment, where it activates immune responses and attracts cytotoxic T cells. Concurrently, it suppressed apoptosis in tumor cells, bolstered their resilience against oxidative stress from ionizing radiation, and contributed to resistance to radiotherapy.^49^ Shu's^50^ study found that applying triptolide (TPL), an inhibitor of HSP70, alongside radiotherapy significantly reduced P-PI3K and P-Akt expression in U251 glioma cells, thereby increasing their radiotherapy sensitivity. Additionally, an HSP70 inhibitor was shown to interfere with the restoration of DNA damage caused by ionizing radiation. Multiple studies have indicated that combining ionizing radiation with HSP70 inhibitors exacerbates the formation of γ-H2AX foci, increases the number of unrepaired double-trand breaks (DSBs), delays their repair process, and enhances tumor cell radiosensitivity.^51^^,^^52^ These findings underscore the significance of HSPs in tumor radioresistance, supporting the feasibility and necessity of our related research. Despite the limited research on HSPs in gliomas, our innovative findings revealed that HSP90B1 could mediate radioresistance by inhibiting radiotherapy-induced apoptosis. The mechanisms of radiotherapy resistance were complex, involving DNA damage repair, apoptosis, and regulation of the cell cycle.^28^ Our research, from the perspective of apoptosis inhibition, revealed a new mechanism of HSP90B1 in glioma radioresistance, providing new insights into the study of glioma radiotherapy resistance.

We further investigated the specific mechanisms through which HSP90B1 exerts its role, finding that HSP90B1 mediated radiotherapy resistance by inhibiting the degradation of RhoC via the ubiquitin-proteasome pathway. RhoC, a member of the Rho GTPase family, was a small, highly conserved protein critical to cancer progression and metastasis.^53^^,^^54^ Studies have shown that RhoC regulates cell migration, cell cycle progression, and various transcription networks,^55^^,^^56^ promoting the progression of cancers such as breast, stomach, and ovarian cancers.^57^, ^58^, ^59^, ^60^ Consistently, our research also identified the role of RhoC in glioma metastasis and found that HSP90B1 promoted tumor migration and invasion by regulating RhoC expression. Additionally, RhoC's role in modulating tumor radioresistance was noted. RhoC enhanced the radioresistance of cervical cancer cells by acting on its downstream target Rho Associated Coiled-Coil Containing Protein Kinase 2 (ROCK2), and this was pivotal in regulating DNA repair within these cells.^35^ In our study, we also demonstrated the regulatory role of RhoC in glioma radioresistance, revealing that HSP90B1 might promote tumor radioresistance by modulating RhoC expression. Heat shock proteins helped proteins fold correctly, maintained the stability of mutated proteins in tumors, and promoted tumor survival and resistance to treatment.^61^ Innovatively, we found that HSP90B1 could bind directly to RhoC, protecting it from degradation via the ubiquitin–proteasome pathway. However, our investigation into the interaction mechanism between HSP90B1 and RhoC remains limited. Structurally, HSP90B1 functioned as a homodimer composed of monomeric subunits and contained three major conserved domains: the N-terminal domain (NTD), the middle domain (MD), and the C-terminal domain (CTD).^62^^,^^63^ The MD functioned as the primary site for substrate recognition and modulated ATPase activity. Binding of the substrate to this domain resulted in increased enzymatic hydrolysis of ATP.^64^ Based on these findings, we hypothesized that the MD may represent the binding domain between HSP90B1 and RhoC. Further studies shoud be conducted to elucidate the precise molecular mechanism underlying the regulation of RhoC by HSP90B1.

Among the HSP90 family members, two additional isoforms, HSP90α and HSP90β, were present in the cytoplasm.^65^ Similar to HSP90B1, which is localized to the endoplasmic reticulum (ER),^66^ both isoforms shared comparable mechanisms of ATP binding and hydrolysis. However, unlike cytoplasmic HSP90, HSP90B1 primarily interacted with calcium-binding proteins.^67^ At the functional level, HSP90α interacted with hypoxia-inducible factor 1α (HIF-1α),^68^ and its deficiency rendered cells more vulnerable to cell death triggered by hypoxic conditions.^69^ Studies further revealed that triple-targeted deficiency of HSP90α/β and CDC37 attenuated epithelial–mesenchymal transition (EMT) in metastatic oral cancer, suppressed extracellular vesicle (EV)-driven tumorigenesis, and reduced EV-induced M2 polarization of macrophages.^70^ In a separate study, overexpression of HSP90β was observed to promote the proliferation, invasion, and migration of gastric carcinoma cells.^71^ Mechanistically, HSP90α and HSP90β mediated their biological functions through several tumor-related signaling networks involving c-Myc, Akt, and Wnt/β-catenin.^72^ Collectively, these isoforms were essential regulators of angiogenesis, cellular invasiveness, metastatic progression, and EMT.^73^ Distinct from these isoforms, HSP90B1 was up-regulated and activated in response to ER stress triggered by factors such as glucose deprivation and hypoxic conditions.^67^ HSP90B1 functioned alongside GRP78 to preserve protein folding fidelity and maintain quality control.^67^ Moreover, HSP90B1 was identified as a potent activator of dendritic cells (DCs),^74^ with surface expression on tumor cells facilitating DC maturation and initiating T cell-mediated immune responses. Beyond its immunological role, HSP90B1 was also involved in the processing of insulin-like growth factors (IGFs), key mediators of survival signaling in tumor cells.^75^ Suppressing HSP90B1 effectively curtailed the expansion of multiple myeloma in a mouse xenograft model.^76^ Notably, our study revealed that HSP90B1 might be involved in mediating radioresistance in glioma.

However, our research also has certain limitations. Our exploration of the mechanisms by which HSP90B1 promotes tumor radioresistance was not sufficiently in-depth. In Figure 3A–D, our observations revealed that HSP90B1 modulated protein levels within the DNA damage repair pathway. The fundamental importance of DNA damage repair in tumor radioresistance primarily manifests in its activation of repair mechanisms to counteract DNA damage induced by radiotherapy, thereby enhancing tumor cell survival.^31^^,^^77^ Additionally, cell cycle arrest plays a crucial role in radioresistance by halting cell division, providing cells with more time to repair radiation-induced DNA damage, thus reducing cell death and increasing radioresistance.^31^ By exploring different directions, we could more fully explain the mechanisms by which HSP90B1 promotes glioma radioresistance, which is the focus of our ongoing research efforts.

Targeting the HSP90B1-RhoC axis might enhance radiosensitivity by concurrently disrupting proteostasis and inhibiting cell migration. Further studies are required to evaluate the *in vivo* therapeutic efficacy of small-molecule agents such as Grp94 inhibitor-1. In addition, we intended to employ AlphaFold for structural modeling of the HSP90B1 RhoC complex, aiming to delineate the interaction site, identify critical residues, and characterize the spatial arrangement. This strategy is expected to complement the mechanistic interpretation by providing atomic-level insights into the selective stabilization of RhoC by HSP90B1, as opposed to other client proteins. Based on the predicted site, it was possible to rationally design small molecules that could disrupt this interaction without compromising the broader cellular functions of HSP90B1. Furthermore, targeted mutagenesis of high-confidence contact sites was considered to evaluate their contribution to radiation responsiveness, laying a theoretical foundation for the development of HSP90B1–RhoC selective inhibitors with improved efficiency over conventional screening methods.^78^

## Conclusions

Overall, our study revealed that by directly binding to RhoC and protecting it from degradation via the ubiquitin–proteasome pathway, HSP90B1 promoted glioma cell metastasis and radioresistance. Inhibiting HSP90B1 could increase the sensitivity of glioma cells to radiotherapy, offering new insights for adjunctive clinical treatment of glioma.

## CRediT authorship contribution statement

**Jiacheng Xu:** Writing – original draft, Visualization, Validation, Software, Investigation, Formal analysis, Data curation. **Yuduo Guo:** Software, Investigation, Funding acquisition, Formal analysis. **Jingjing Yang:** Investigation. **Guanjie Shang:** Investigation. **Weihai Ning:** Investigation. **Deshan Liu:** Investigation. **Hongwei Zhang:** Writing – review & editing, Supervision, Resources, Project administration, Methodology, Funding acquisition. **Yongmei Song:** Writing – review & editing, Supervision, Resources, Project administration, Methodology, Funding acquisition.

## Ethics declaration

The study received authorization from the Ethics Committee of Sanbo Brain Hospital, Capital Medical University (SBNK-YJ-2020-001-01). The mouse study was approved by the Biosafety, Ethics, and Experimental Animal Management Committee of the Institute of Biophysics, Chinese Academy of Sciences, with the approval number SYXK2021124.

## Data availability

Access to the data and code used in this research was provided by the corresponding author upon reasonable inquiry.

## Funding

The National Natural Science Foundation of China (No. 82101932) financed this research.

## Conflict of interests

No commercial or financial interests influenced this study, as confirmed by the authors, ensuring no potential conflicts of interest.
