# Supplementary material for: HSP90B1 facilitates glioma radiotherapy resistance by regulating RhoC ubiquitin‒proteasome degradation
Source: Genes Dis. 2025 Jul 1;13(3):101756. doi: 10.1016/j.gendis.2025.101756 (PMC12914105; doi:10.1016/j.gendis.2025.101756)
Supplement: Multimedia component 1 [file mmc1.docx]

**Supplementary Figures**

**
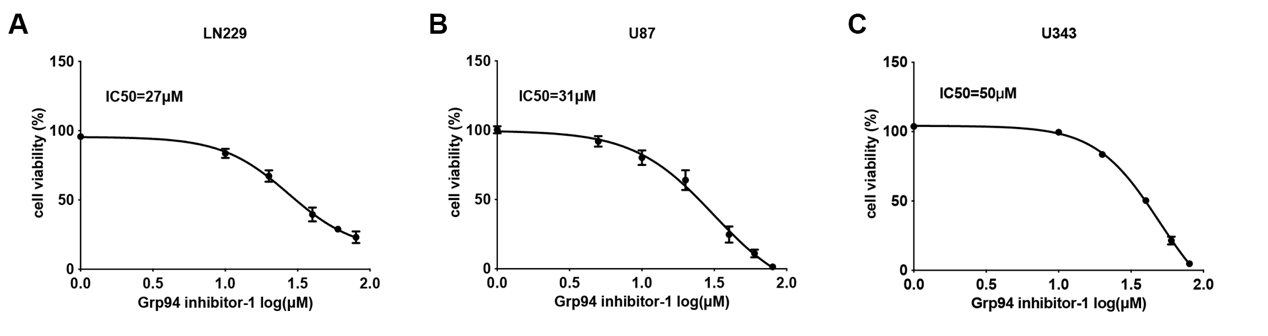
**

**Supplementary Figure 1. Proliferation inhibition curves and determination of IC50 values for different cells in response to varying concentrations of HSP90B1 inhibitor.** Using the CCK8 assay, survival curves of LN229 (A), U87 (B), and U343 (C) cells were plotted under treatment with different concentrations of Grp94 inhibitor-1. The IC50 values were determined after curve fitting.


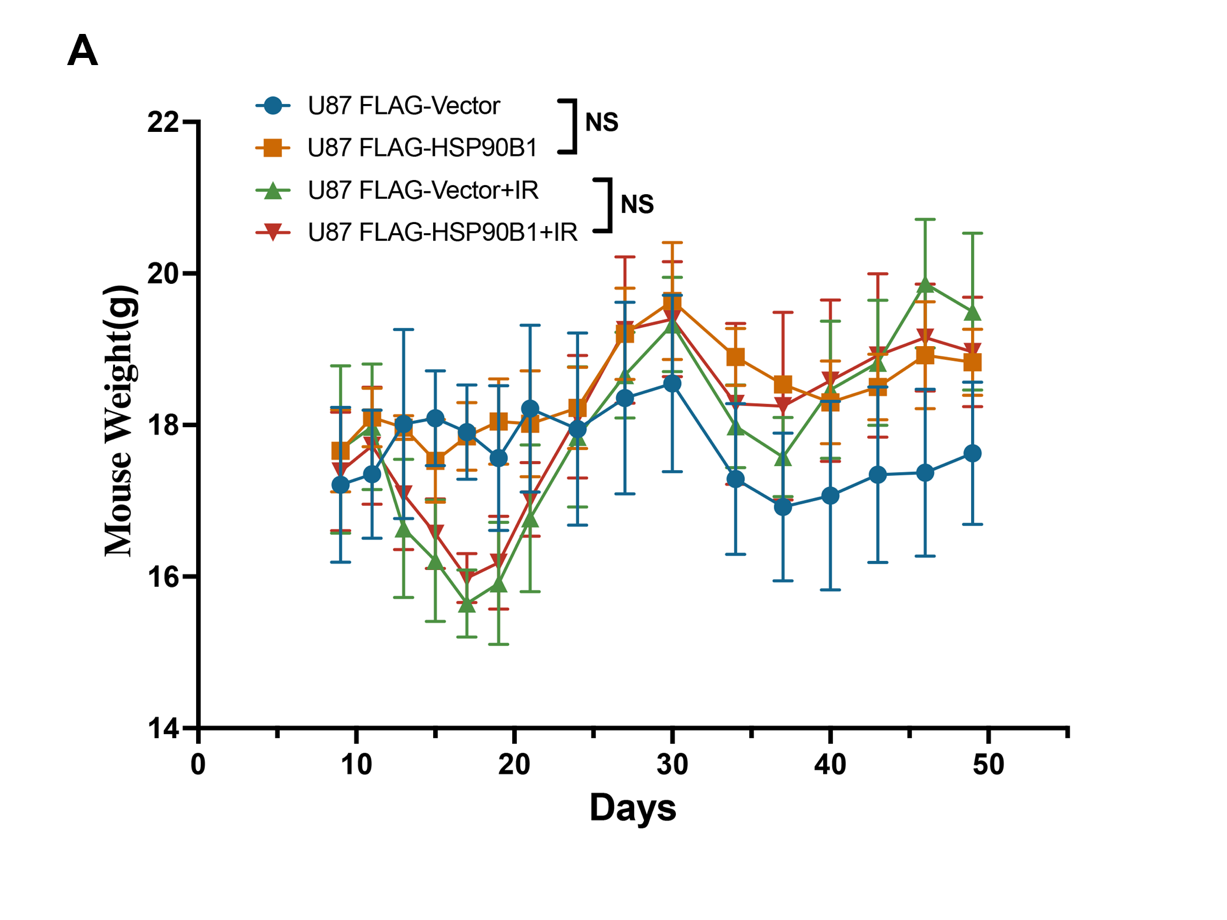


**Supplementary Figure 2. Mouse weight change curves.** A. The experiment was divided into four groups: Flag-Vector, Flag-HSP90B1, Flag-Vector + IR, and Flag-HSP90B1 + IR. Changes in mouse weight were monitored across the different treatment groups.
