# Supplementary material for: HSP90B1 facilitates glioma radiotherapy resistance by regulating RhoC ubiquitin‒proteasome degradation
Source: Genes Dis. 2025 Jul 1;13(3):101756. doi: 10.1016/j.gendis.2025.101756 (PMC12914105; doi:10.1016/j.gendis.2025.101756)
Supplement: Multimedia component 2 [file mmc2.docx]

**Supplementary Tables**

**Supplementary table 1: Sequences of target gene-specific primers.**

| **Primers** | **Sequences** |
| --- | --- |
| HSP90B1-Forward primer  HSP90B1-Reverse primer | ACTGATTTCAAATGCTTCTGATGCT  CAGGTTCTTCTCCTTATCACACTTA |
| RhoC-Forward primer  RhoC-Reverse primer | CGGAGCGGAAGCCTTGAC  CCCAACGATCACCAGCTTCT |
| ACTIN-Forward primer  ACTIN-Reverse primer | CTGGCTCCTAGCACCATGAAGAT  GGTGGACAGTGAGGCCAGGAT |

**Supplementary table 2: Detail sequences of the siRNAs purchased.**

| HSP90B1 siRNAs | Sequences |
| --- | --- |
| HSP90B1-1921(si2) | 5’-GGAGAGUCGUGAAGCAGUUTT-3’,  5’-AACUGCUUCACGACUCUCCTT-3’ |
| HSP90B1-2182(si3) | 5’-GCUGAUCAGAGACAUGCUUTT-3’, 5’-AAGCAUGUCUCUGAUCAGCTT-3’ |

**Supplementary table 3: Detail sequences of shHSP90B1.**

|  | Sequences |
| --- | --- |
| shHSP90B1 | 5’-GGAGAGUCGUGAAGCAGUUTT-3’,  5’-AACUGCUUCACGACUCUCCTT-3’ |

**Supplementary table 4: Detailed information on the antibodies used in the study.**

| **Antibodies** | **Manufacturer** |
| --- | --- |
| HSP90B1 antibody | Proteintech, 14700-1-AP |
| RhoC antibody | Proteintech, 67542-1-Ig |
| RAD50 antibody | Cell Signaling Technology, 3427T |
| pH2Ax antibody | Cell Signaling Technology, 9718T |
| Bcl-2 antibody | Santa, sc-7382 |
| Bax antibody | Cell Signaling Technology, 2772S |
| Caspase-3 antibody | Cell Signaling Technology, 9662S |
| Cleaved Caspase-3 antibody | Cell Signaling Technology, 9664T |
| PARP/Cleaved-PARP antibody | Proteintech, 13371-1-AP |
| Flag antibody | Cell Signaling Technology, 14793T |
| Ubiquitin antibody | Proteintech, 10201-2-AP |
| β-Actin antibody | Cell Signaling Technology, 3700S |

**Supplementary table 5: Detailed information on the reagents used in the study.**

| **Reagents** | **Manufacturer** |
| --- | --- |
| Lipofectamine 2000 | Invitrogen(Shanghai, China) |
| NEOFECT DNA transfection reagent | Neofect (Beijing, China) biotech Co.,Ltd |
| Fetal Bovine Serum (FBS) | XiGong Biotechnology (Beijing, China) |
| DMEM medium | XiGong Biotechnology (Beijing, China) |
| Trypsin digestive fluid | New Cell & Molecular Biotech Co.,Ltd(Suzhou, China) |
| Total RNA column extraction kit | New Cell & Molecular Biotech Co.,Ltd(Suzhou, China) |
| Grp94 Inhibitor-1 | MedChemExpress(Shanghai, China) |
| Annexin V-FITC/PI apoptosis assay kit | Keygen Biotech Corp.,Ltd(Jiangsu, China) |
| Annexin V-APC/PI apoptosis assay kit | Keygen Biotech Corp.,Ltd(Jiangsu, China) |
| Protein A/G PLUS-Agarose | Santa Cruz Biotechnology (Shanghai, China) Co., Ltd |
| Cell Counting Kit-8 | New Cell & Molecular Biotech Co.,Ltd(Suzhou, China) |
| Reverse Transcription Kit | Promega(Beijing, China)Biotech Co.,Ltd |
| Protease Inhibitor Cocktail | New Cell & Molecular Biotech Co.,Ltd(Suzhou, China) |
| RIPA Lysis Buffer | Applygen Technologies Inc.(Beijing, China) |
| Cycloheximide | MedChemExpress(Shanghai, China) |
| MG132 | GlpBio(Shanghai, China) |
| BCA Protein Assay Kit | New Cell & Molecular Biotech Co.,Ltd(Suzhou, China) |
| NcmECL Ultra | New Cell & Molecular Biotech Co.,Ltd(Suzhou, China) |
| GoldHi EndoFree Plasmid Maxi Kit | Jiangsu Cowin Biotech Co., Ltd(Jiangsu, China) |
| Protein Stains O | Sangon Biotech (Shanghai, China) Co., Ltd |
